# Staufen1 Regulates Multiple Alternative Splicing Events either Positively or Negatively in DM1 Indicating Its Role as a Disease Modifier

**DOI:** 10.1371/journal.pgen.1005827

**Published:** 2016-01-29

**Authors:** Emma Bondy-Chorney, Tara E. Crawford Parks, Aymeric Ravel-Chapuis, Roscoe Klinck, Lynda Rocheleau, Martin Pelchat, Benoit Chabot, Bernard J. Jasmin, Jocelyn Côté

**Affiliations:** 1 Department of Cellular and Molecular Medicine, University of Ottawa; Centre for Neuromuscular Disease, Ottawa, Ontario, Canada; 2 Département de microbiologie et d'infectiologie, Université de Sherbrooke, Sherbrooke, Québec, Canada; 3 Department of Biochemistry, Microbiology and Immunology, University of Ottawa, Ottawa, Ontario, Canada; The Jackson Laboratory, UNITED STATES

## Abstract

Myotonic dystrophy type 1 (DM1) is a neuromuscular disorder caused by an expansion of CUG repeats in the 3' UTR of the *DMPK* gene. The CUG repeats form aggregates of mutant mRNA, which cause misregulation and/or sequestration of RNA-binding proteins, causing aberrant alternative splicing in cells. Previously, we showed that the multi-functional RNA-binding protein Staufen1 (Stau1) was increased in skeletal muscle of DM1 mouse models and patients. We also showed that Stau1 rescues the alternative splicing profile of pre-mRNAs, e.g. the *INSR* and *CLC1*, known to be aberrantly spliced in DM1. In order to explore further the potential of Stau1 as a therapeutic target for DM1, we first investigated the mechanism by which Stau1 regulates pre-mRNA alternative splicing. We report here that Stau1 regulates the alternative splicing of exon 11 of the human *INSR* via binding to Alu elements located in intron 10. Additionally, using a high-throughput RT-PCR screen, we have identified numerous Stau1-regulated alternative splicing events in both WT and DM1 myoblasts. A number of these aberrant ASEs in DM1, including *INSR* exon 11, are rescued by overexpression of Stau1. However, we find other ASEs in DM1 cells, where overexpression of Stau1 shifts the splicing patterns away from WT conditions. Moreover, we uncovered that Stau1-regulated ASEs harbour Alu elements in intronic regions flanking the alternative exon more than non-Stau1 targets. Taken together, these data highlight the broad impact of Stau1 as a splicing regulator and suggest that Stau1 may act as a disease modifier in DM1.

## Author Summary

Myotonic Dystrophy Type 1 (DM1) is an inherited disorder affecting many systems, including skeletal muscle, heart, eyes and endocrine system. DM1 is known as a ‘trinucleotide repeat disorder’ because it is caused by an abnormal expansion of a highly repeated motif within the *DMPK* locus. Such an expansion results in the expression of a ‘toxic RNA’, which causes misregulation of proteins involved in many essential cellular pathways. Research efforts have largely focused on misregulation of a very few splicing regulators that can be linked with many defects observed in the pathology. We have recently uncovered that the multifunctional RNA-binding protein Staufen1 is increased in DM1, and that it is capable of rescuing selected defects in DM1 cells, including alternative splicing of the *INSR* pre-mRNA, which is linked with insulin resistance. Given the potential impact of this novel function for Staufen1, we investigated the mechanism by which it regulates splicing, and determined that it mediates its effects through binding to conserved genomic repetitive sequences called Alu elements. We also uncovered that Staufen1 influences the splicing of numerous genes in DM1 patient cells, predictive to either improve or worsen the pathology, thus identifying Staufen1 as a novel disease modifier in DM1.

## Introduction

Alternative splicing of pre-mRNAs is a phenomenon allowing multiple mRNA transcripts to be produced from a single pre-mRNA. Recent reports estimate that 95–100% of human multi-exon genes produce two or more mRNA splice variants, with a majority yielding an average of eight variants [1–5]. Generation of these variants by alternative splicing is a major mechanism responsible for the complexity of the transcriptome and proteome observed in eukaryotes [6]. Constitutive RNA splicing occurs through the recognition of the core splicing signals: the 5' splice site, branch point, polypyrimidine tract, and the 3' splice site AG by the spliceosome components. Additional cis-regulatory elements including exonic splicing silencers (ESS) and enhancers (ESE), and intronic splicing silencers (ISS) and enhancers (ISE) can influence the usage of core splicing signals. Moreover, intronic elements resembling splice sites can act as "decoy" splice sites to influence alternative splicing [7]. There is also a variety of conserved RNA secondary structures that interfere with the recognition of splicing signals and influence splice site selection [8, 9]. The binding of these regulatory elements by RNA-binding proteins can inhibit or enhance the use of core splice sites and results in alternative splicing. Regulation of alternative splicing is thus mediated through the intricate interplay between these cis-acting and trans-acting regulatory elements.

Deciphering the mechanisms that regulate alternative splicing is essential for understanding how cellular diversity and specialization are generated but, importantly, it is also critical to develop novel therapeutic approaches for a growing number of diseases caused by misregulation of pre-mRNA splicing ([10] and refs. therein). For example, in the neuromuscular disorder Myotonic Dystrophy Type 1 (DM1), an expansion of CTG repeats in the 3'UTR of the Dystrophia Myotonica Protein Kinase (*DMPK*) gene results in retention of CUG-containing DMPK mRNAs within specific RNA foci in the nucleus. The length of CTG repeats varies in DM1 patients and correlates with disease severity [11]. The mutant CUG-expanded mRNA causes a large misregulation of many splicing factor proteins, such as MBNL1, CUGBP1, hnRNP H, ASF/SF2 and RBFOX1 (for review see [12–14]). The misregulation of these splicing factors is reflected in the splicing defects observed in the DM1 pathology. Previously, it has been reported that 13 aberrant splicing events could be linked with the complex DM1 phenotype [15, 16], but more recent studies suggest the existence of numerous additional splicing defects in DM1 tissues [17–19]. One important example of mis-splicing in DM1 is the increase in exon 11 exclusion of the insulin receptor (*INSR)*, which results in the overproduction of the IR-A splice variant, thereby contributing to insulin resistance in DM1 patients [20]. A variety of splicing factors acting on multiple cis-regulatory elements contribute to the splicing control of *INSR* and of other alternative splicing events. Indeed, a recent report suggests that CUGBP1 and MBNL1 antagonistically regulate hundreds of alternative exons and compete for binding to specific pre-mRNAs [21].

Stau1 is a highly conserved multi-functional double-stranded RNA-binding protein involved in key aspects of RNA metabolism. These include mRNA transport and localization, translation efficiency, stability, regulation of stress granule assembly, and both nuclear and unconventional cytoplasmic mRNA alternative splicing [22–32]. In mammals, Stau1 pre-mRNA is alternatively spliced to produce two major forms Stau1^55^, Stau1^63^ and one variant reported to not bind RNA, Stau1^i^ [24, 33, 34]. Recently, several high-profile studies have focused on elucidating Stau1 binding sites (SBS), which are crucial for understanding Stau1’s ability to regulate mRNA metabolism [31, 35–37]. Extensive work by numerous groups has utilized various immunoprecipitation techniques to investigate SBS, which appear to be represented by a highly diverse group of RNA secondary structures. These include double-stranded RNA structures containing stems and motifs ranging in size from 5–22 base pairs (bps) to hundreds of bps long which, in turn, can contain multiple short binding sites with varying degree of perfect base pairing, displaying little to no sequence specificity [31, 35–37]. Notably, in all large-scale studies performed to date, Stau1 has been reported to bind preferentially to the primate-specific, mobile element called Alu elements. SBS, comprised from both Alu and non-Alu element containing sequences, have been found everywhere in the genome including 3'UTRs, 5'UTRs, intronic regions, coding sequences and intergenic regions [31]. This diversity of SBS location highlights the potential complexity surrounding events regulated by Stau1.

Recently, our group identified Stau1 as being significantly increased in muscle samples from adult DM1 patients and DM1 mouse models [32]. Additionally, we saw that further overexpression of Stau1 in DM1 was able to rescue key hallmarks of the pathology, such as increased export and translation of CUG-expanded mRNAs and a significant increase in *INSR* exon 11 inclusion [32]. Interestingly, our study revealed for the first time the ability of Stau1 to regulate alternative pre-mRNA splicing suggesting a novel role for Stau1 as a splicing regulator [32]. These data lead us to speculate that the upregulation of Stau1 represents a positive and protective adaptation in the DM1 pathology.

Here, we first set out to determine the mechanism by which Stau1 regulates pre-mRNA alternative splicing. Second, we examined the broader impact of Stau1 as a splicing regulator in the context of DM1. We report that Stau1 regulates the alternative splicing of human *INSR* exon 11 via binding to a region harbouring Alu elements within intron 10. Additionally, using a high-throughput RT-PCR screen, we identified numerous Stau1-regulated Alternative Splicing Events (ASEs) in both WT and DM1 myoblasts. These Stau1-induced changes in ASEs are expected to be beneficial or detrimental for the DM1 pathology. Importantly, a higher number of Stau1-regulated ASEs harbour Alu elements in intronic regions flanking the alternative exon when compared to non-Stau1 ASE targets. We thus propose that Stau1 uses Alu elements to regulate a large set of ASEs and that it acts as a disease modifier impacting on the severity of DM1.

## Results

### Human Insulin Receptor (*INSR*) alternative exon 11 inclusion is regulated by Stau1

**Fig 1:**
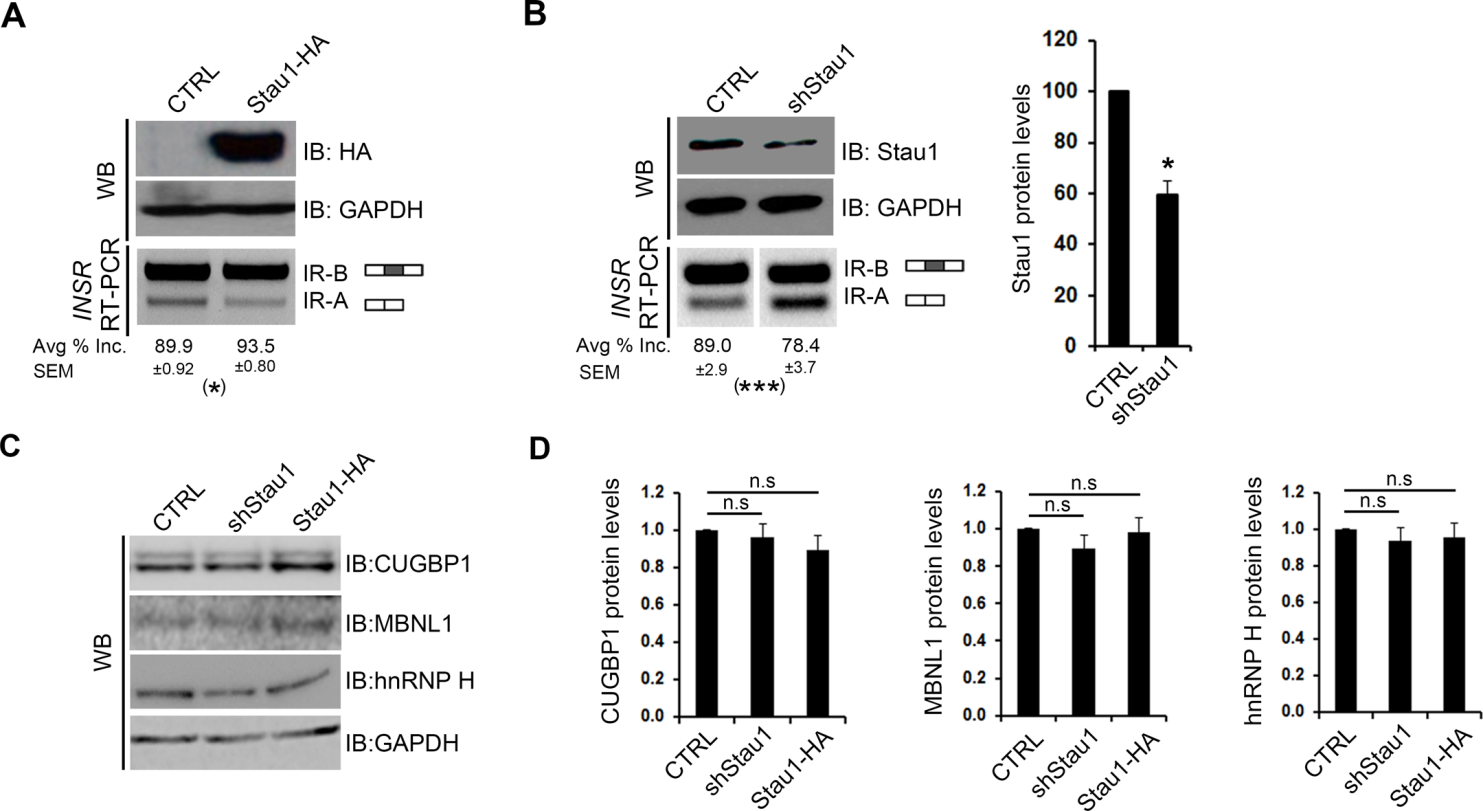
Stau1 levels regulate the pre-mRNA splicing of the human *INSR* in HeLa cells. (A) pGIPZ (CTRL) or Stau1-HA (Stau1-HA) plasmids were transiently transfected into HeLa cell lines, and total RNA and protein lysate was collected after 48 hours. RT-PCR using primers specific to the human endogenous *INSR* were used on cDNA synthesized from total RNA to amplify the two isoforms (IR-A and IR-B) of the *INSR*. Stau1-HA protein levels were assessed by Western blot using HA-specific antibodies, and GAPDH was used as a loading control. (B) shCTRL or shStau1 were transiently transfected into HeLa cell lines and total RNA and protein lysate was collected after 48 hours. RT-PCR was performed to amplify the *INSR* isoforms. Stau1 protein levels were assessed by Western blot and quantified using GAPDH as a loading control. (C) Representative Western blots showing protein levels of CUGBP1, MBNL1 and hnRNP H in HeLa cells transfected with CTRL, shRNA or Stau1-HA plasmids. GAPDH was used as a loading control. (D) Quantification of Western blot analysis of splicing factors upon Stau1 level modulation. In all cases, bar graphs show an average of ≥3 independent experiments. Error bars represent SEM * = p < 0.05, ** = p < 0.01.

We recently reported that Stau1 overexpression rescues specific alternative splicing defects associated with DM1, including that of exon 11 in the *INSR* pre-mRNA [32]. Moreover, our observation that Stau1 also promotes exon 11 inclusion in muscle cells in the absence of pathological RNA repeats suggests that Stau1 may be a bona fide splicing regulator. In order to explore this idea further, we first assessed whether Stau1 could affect *INSR* exon 11 alternative splicing in non-muscle cells. First, HeLa cells were transiently transfected with a Stau1-HA expression construct and the relative level of endogenous *INSR* exon 11 inclusion was determined using semi-quantitative RT-PCR. A high level of exon 11 inclusion was observed in these cells which agrees with previous findings [38]. Similar to our previous work in C2C12 myoblasts [32], the overexpression of Stau1-HA, as confirmed by Western blotting with HA-antibodies, resulted in a small, but reproducible ~5% increase in exon 11 inclusion (Fig 1A). To address whether Stau1 is required to maintain normal levels of *INSR* exon 11 inclusion, we also assessed *INSR* splicing event when levels of Stau1 are reduced. This would also mitigate the possibility that our observed effects on splicing were due to spurious, non-specific RNA binding of overexpressed Stau1-HA. HeLa cells were thus transiently transfected with a Stau1-targeting shRNA mix (described in materials and methods) and Western blotting was performed to assess Stau1 protein levels. This analysis demonstrated a 40% reduction of Stau1 protein level compared to CTRL (Fig 1B). This reduction of Stau1 levels caused a significant ~10% decrease in the relative inclusion of *INSR* exon 11 (Fig 1B). To confirm further the role of Stau1 as a splicing regulator in non-muscle cells, we extended our work to include an additional cell line, namely, HEK293Ts. In agreement with our findings in HeLa cells, the overexpression and reduction of Stau1 resulted in a significant ~5% increase and ~10% decrease, respectively, of exon 11 inclusion (S1A Fig).

The splicing of exon 11 is known to be regulated by a number of splicing factors, including, but not limited to MBNL1, CUGBP1, and hnRNP H [39–41]. These splicing proteins, similar to Stau1, are misregulated in DM1 [20, 42–44]. Thus, it was important to determine if our data are indicative of a direct effect of Stau1 on exon 11 splicing regulation or an indirect effect mediated through modified expression of other splicing regulators. In both HeLa and HEK293T cells, no significant changes were observed in the mRNA or protein levels of MBNL1, CUGBP1 and hnRNP H, upon modulation of Stau1 levels (Fig 1C and 1D and S1B Fig and S1C Fig). Finally, as we have previously confirmed, Stau1 over-expression did not differentially affect the mRNA half-lives of *INSR* alternatively spliced variants [32]. Thus, altogether, our results strongly suggest that Stau1 is a bona fide splicing regulator, participating in the maintenance of human *INSR* exon 11 alternative splicing profile.

### Stau1 regulates splicing of *INSR* exon 11 through an interaction with Alu elements in intron 10

**Fig 2:**
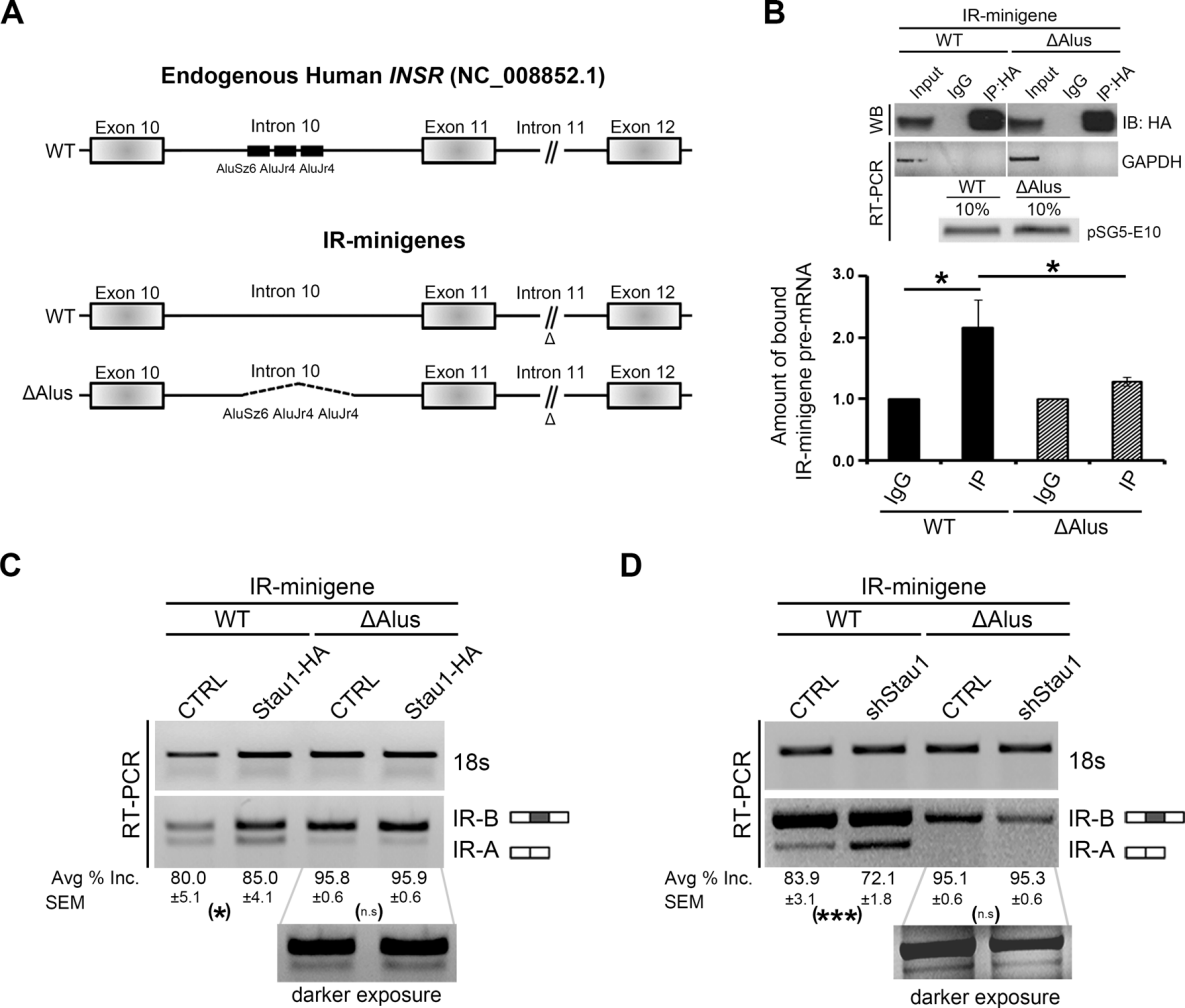
Stau1 regulates splicing of *INSR* exon 11 through an interaction with Alu elements in intron 10. (A) The genomic DNA sequence of the human INSR (NG_008852.1) was used to assess the Alu elements located in intron 10. Introns are not to scale, and this is indicated in intron 11 (//). The IR-minigene constructs used in this study are shown here. Previously deleted segments of genomic DNA determined not to influence exon 11 splicing are indicated in intron 11 (Δ symbol), and black dotted lines represent the deleted segment containing the three Alu elements. (B) HeLa cells were transiently transfected with Stau1-HA plasmid and either the WT or ΔAlus IR-minigene. Immunoprecipitation (IP) of Stau1-HA protein was carried out using HA-specific antibodies under RNase-free conditions. Western blot using HA-antibodies show equal amounts of Stau1-HA protein was immunoprecipitated in each condition. RNA was collected and DNase-treated prior to cDNA synthesis. RT-PCR was performed using *GAPDH* specific primers to demonstrate a lack of non-specific binding of RNA to the beads used for immunoprecipitation. Equal amounts of transfected minigenes were confirmed by performing RT-PCR on the cDNA synthesized from the 10% inputs lysates with primers specific to amplify a portion of the plasmid vector (pSG5) and the IR-minigene (Exon 10), corresponds to the pSG5-E10 labelled band. RT-qPCR was carried out using primers specific to an 115 bp region of intron 10 of the IR-minigene to determine the amount of IR-minigene RNA bound to immunoprecipitated Stau1-HA. Bar graphs show an average of four independent RIP experiments. (C-D) HeLa cells were transiently transfected with a CTRL, Stau1-HA plasmid or shStau1 and either the WT or ΔAlus IR-minigene. IR-minigene splicing was determined by RT-PCR. The average of ≥3 independent experiments was used. Error bars represent SEM * = p < 0.05, ** = p < 0.01, *** = p < 0.001.

In order to gain insights into the mechanism by which Stau1 regulates *INSR* exon 11 alternative splicing, we first searched for possible SBSs, which could represent cis-regulatory elements within the *INSR* pre-mRNA. Recently, numerous reports have emerged describing SBSs and although no single SBS has been described, one of the most highly recurring SBS reported is composed of Alu Repeat elements [31, 35–37, 45, 46]. Interestingly, an earlier report described the presence of an Alu Repeat element located in intron 10 of the *INSR* [47]. Closer inspection of this region via a bioinformatic analysis revealed that, in fact, there are three Alu elements located upstream of the intron 10-exon 11 boundary (Fig 2A). The fact that Alu elements are preferred SBS [45], together with the presence of Alu elements in intron 10 of the *INSR*, led us to propose that Stau1 may bind to these Alu elements to regulate alternative splicing of exon 11. To test this hypothesis, we selected two IR-minigene constructs [40, 41, 47]: WT and ΔAlus (in which all three Alu elements are deleted) (Fig 2A). HeLa cells were co-transfected with Stau1-HA and either the WT or the ΔAlus IR-minigene. RIP experiments were performed on cell lysates and RT-qPCR using primers specific for the intronic sequence of intron 10 was performed to identify the amount of IR-minigene pre-mRNA bound to Stau1-HA (Fig 2B). The amount of Stau1-HA bound to WT IR-minigene demonstrated a significant ~2-fold enrichment over the IgG control (Fig 2B; black bars). This degree of association was greatly reduced with the ΔAlus IR-minigene (Fig 2B; crosshatch bars), consistent with the hypothesis that Stau1 binds to the pre-mRNA of the *INSR* via the Alu elements located in intron 10.

Next, we investigated whether the Alu elements were necessary for Stau1-regulated splicing of exon 11 by carrying out Stau1 overexpression and knockdown experiments. First, HeLa cells were co-transfected with a Stau1-HA expression or shStau1 construct and one of the IR-minigenes; WT or ΔAlus. Overexpression of Stau1-HA induced an ~5% increase in exon 11 inclusion in the WT IR-minigene (Fig 2C), as determined by RT-PCR as above. The reduction in Stau1 led to an ~12% decrease in exon 11 inclusion in the WT IR-minigene (Fig 2D). Importantly, in the absence of the Alu elements in the IR-minigene, neither overexpression nor reduction of Stau1 resulted in a significant change in exon 11 inclusion (Fig 2C and 2D, respectively). Thus, our data demonstrate that the Alu elements located in intron 10 are essential for Stau1 splicing regulation of exon 11 of the IR-minigene.

### High-throughput RT-PCR reveals that Stau1 regulates the alternative splicing of numerous mRNAs in both WT and DM1 conditions

The observation that Stau1 regulates the splicing of the *INSR* led us to examine whether Stau1 regulates the splicing of additional pre-mRNAs. To address this central question, we carried out a screen using a high-throughput RT-PCR screen that measured the changes in the splicing ratios of 487 selected events (as described in [18]) in MyoD-converted WT and DM1 myoblasts either overexpressing GFP or Stau1-HA. The 487 ASEs comprising the RT-PCR alternative splicing screen were chosen based on their association with the specific key terms: "muscle", "glucose metabolism", "wasting", and "ion-channel". WT (GM03377) or DM1 fibroblasts harbouring 1700 CTG (GM03132) repeats in the 3'UTR of the *DMPK* gene, were converted to myoblasts. Briefly, the conversion was done via two rounds of infection over 48 hours with a retrovirus engineered to express MyoD, followed by selection with Puromycin (1 μg/mL) for 5 days. Cultures were then infected with either GFP- or human Stau1-HA lentiviral particles. GFP expression was used to confirm infection efficiency 48 hours post-infection (S2A Fig). Semi-quantitative RT-PCR and Western blot analysis using *MyoD* specific primers and MyoD antibodies confirmed the overexpression of *MyoD* mRNA and protein (S2B Fig and S2C Fig). Moreover, Western blot analysis with anti-HA tag antibodies confirmed Stau1-HA overexpression (S2D Fig). Total RNA from the MyoD-converted WT and DM1 cells was then isolated and used to carry out the high-throughput RT-PCR splicing screen.

**Fig 3:**
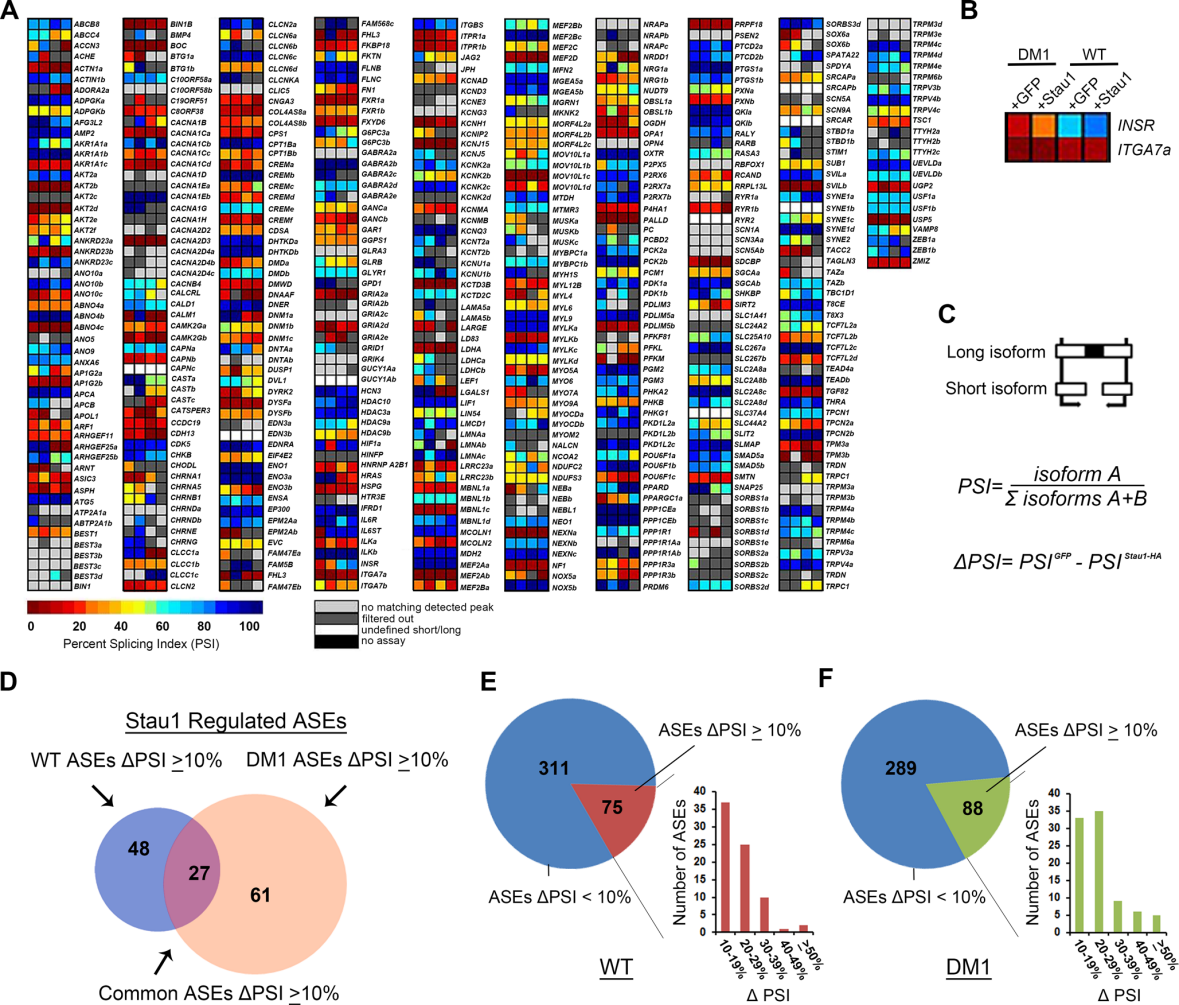
Analysis of high-throughput RT-PCR splicing screen. (A) Raw data output displayed as heatmap from RT-PCR splicing screen. A total of 489 ASEs were analysed in the high-throughput RT-PCR Screen. Any ASEs that showed a blank reading for Percent Splicing Index (PSI) in any of the four conditions was excluded. (B) Enlarged image of two ASEs in DM1 and WT conditions. (C) PSI represented here as a ratio between the long isoform and the short isoform. The changed in PSI (ΔPSI) was calculated for each ASE in both conditions. (D) Total number of ASEs that showed a PSI change ≥10% with Stau1-HA overexpression in WT and DM1. (E-F) Stau1 and non-Stau1 (<10% ΔPSI) regulated ASEs. Bar graphs display the ΔPSI groups (according to percent change) of Stau1-regulated ASEs in both WT and DM1.

Initial heat map data from the RT-PCR screen revealed that the overexpression of Stau1 in both WT and DM1 conditions had a broad effect on the splicing profile of numerous ASEs through the observed alteration of the Percent Splicing Index (PSI) for each ASE (Fig 3A and 3B). Although several ASEs were not affected by Stau1-HA overexpression (e.g. *ITGA7a*; Fig 3B), a number of ASEs showed important changes in both WT and DM1 conditions (e.g. *INSR*; Fig 3B). To determine quantitatively whether Stau1 increase regulates the PSI of an ASE, Stau1-HA overexpression was compared to GFP CTRL from WT and DM1 cell lines, yielding a value referred to as the change in PSI (ΔPSI). A threshold of a ΔPSI ≥10% was established to denote relevant changes in splicing regulated by Stau1 (Fig 3C and 3D, S1 Table). Altogether, data from the high-throughput RT-PCR screen demonstrate that overexpression of Stau1-HA affects the splicing of 75 and 88 ASEs in WT and DM1 cell lines, respectively, with 27 ASEs common in both conditions (Fig 3D). Similar trends were seen in both WT and DM1 conditions where the majority of Stau1-regulated ASEs showed a ΔPSI between 10–30% upon Stau1-HA overexpression. Accordingly, only a few ASEs showed large (≥50%) ΔPSI when Stau1-HA was overexpressed, compared to GFP CTRL, such as *INSR and NRG1* (Fig 3E and 3F). These data suggest that Stau1 does not dramatically alter the PSI of the majority of Stau1-regulated ASEs, but instead appears to fine-tune the alternative splicing of many ASEs. These results confirm our hypothesis that Stau1 is a splicing regulator and further show that Stau1 levels can alter the splicing profile of numerous pre-mRNAs both in WT and DM1 conditions.

### ASEs regulated by Stau1-HA overexpression are both potentially beneficial and detrimental for the DM1 pathology

**Fig 4:**
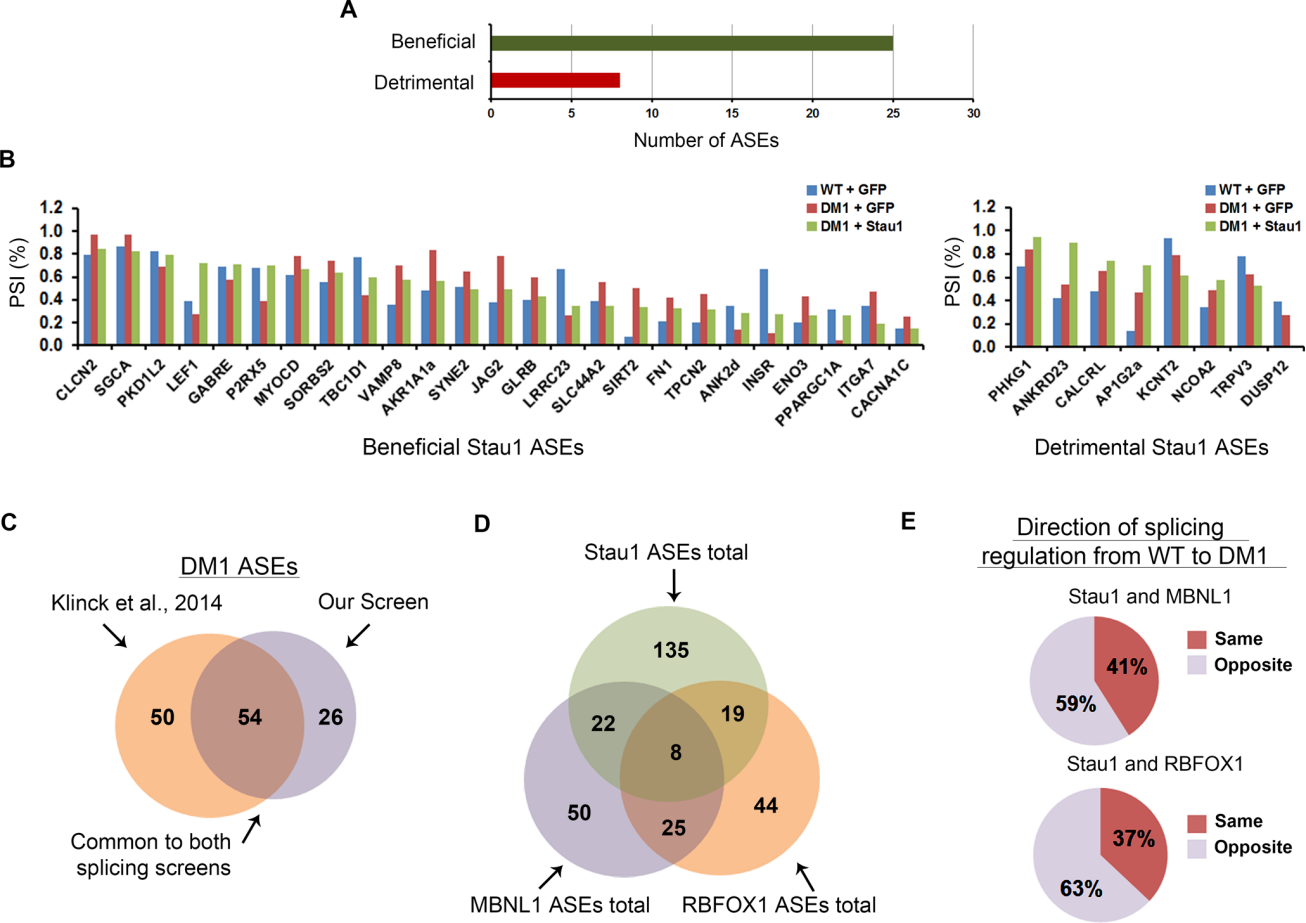
Stau1 overexpression regulates the alternative splicing of numerous ASEs which are both beneficial and detrimental in DM1 conditions. (A) A total of 33 ASEs were considered for this analysis based on the following criteria. First, ASEs that showed a ≥10% ΔPSI from WT to DM1 conditions were selected. From this, ASEs that showed a ≥10% ΔPSI with Stau1 overexpression were selected and identified as either beneficial or detrimental for the DM1 pathology (refer to results). (B) Bar graphs showing the specific ASEs that the overexpression of Stau1-HA under DM1 conditions either returned towards the WT splicing pattern (left bar graph) or continued towards a detrimental effect (right bar graph). (C-E) Comparison of the data obtained from the RT-PCR splicing screen presented here to that of Klinck et al., 2014. The threshold used by Klinck et al., was applied (≥5% PSI) to compare the 163 ASEs used in both screens (Refer to S1 Table). (E) The direction of splicing was determined by identifying whether modification of the splicing proteins (i.e. Stau1 and/or MBNL1/RBFOX1) shifted the splicing pattern of an ASE in similar directions, for example, modification of both splicing factors resulted in an increased splicing of the long isoform.

We recently demonstrated that overexpression of Stau1 in DM1 conditions induced an increased inclusion of the *INSR* exon 11 [32]. This splicing modulation should be beneficial for DM1 as it reverts the *INSR* aberrant splicing towards WT conditions, an event that would also be predicted to reduce insulin resistance in patients. This prompted us to investigate whether the splicing events regulated by Stau1 overexpression were all beneficial for the DM1 pathology. For this analysis, only ASEs which showed a change in splicing pattern ≥10% from WT to DM1 conditions were considered. ASEs that shifted back towards WT splicing patterns when Stau1-HA was overexpressed were considered beneficial. Conversely, an ASE was considered detrimental if the overexpression of Stau1-HA in DM1 conditions exacerbated the splicing pattern observed in the pathology, i.e. opposite direction of WT. Using these criteria, 25 ASEs were classified as beneficial, whereas 8 ASEs would be potentially detrimental upon Stau1-HA overexpression in DM1 patient cells (Fig 4A and 4B). This suggests that promoting Stau1-regulated splicing in DM1 could potentially have both beneficial and detrimental effects depending on the specific alternative splicing event considered. Taken together, these results demonstrate that Stau1 is a splicing factor that regulates a broad range of splicing events and highlights the importance of Stau1 as a potential disease modifier for DM1.

We extended the results collected from our RT-PCR splicing screen by generating a comparison between ASEs altered in DM1 identified in the current study relative to those documented in a recent report by Klinck and colleagues [18]. Using the same thresholds set by Klinck et al., we identified 54 ASEs that appeared altered in the DM1 conditions that were common to both screens, including previously described events such as the *INSR*, *ANK2* and various chloride channels (Fig 4C and S1 Table). The ASEs that were distinct between the two screens may be due to the different cell lines used in each independent study. More specifically, normal adult satellite muscle cells were used in the study by Klinck *et al*., and MyoD-converted myoblast cell cultures were used in our study. These differences in cell culture models may account for the variability in the DM1 associated ASEs identified. We also compared the ASEs regulated by Stau1-HA, to ASEs regulated by MBNL1 or RBFOX1 described by Klinck and colleagues. A total of 22 and 19 ASEs were identified that appeared to be co-regulated by either Stau1 and MBNL1 or RBFOX1, respectively (Fig 4D and S1 Table). A total of eight ASEs were identified as being co-regulated by all three splicing proteins, including *INSR*. A comparison of the direction of splicing regulation of Stau1 to either MBNL1 or RBFOX1 revealed that 51% and 63% of the ASEs co-regulated by Stau1 and MBNL1 or RBFOX1, respectively, proceeded in the same direction (Fig 4E). The fact that Stau1 regulates the same splicing events as MBNL1 and/or RBFOX1 suggests that Stau1 may act as both an agonist and antagonist to other splicing factors.

### Validation of Stau1-regulated splicing targets

**Fig 5:**
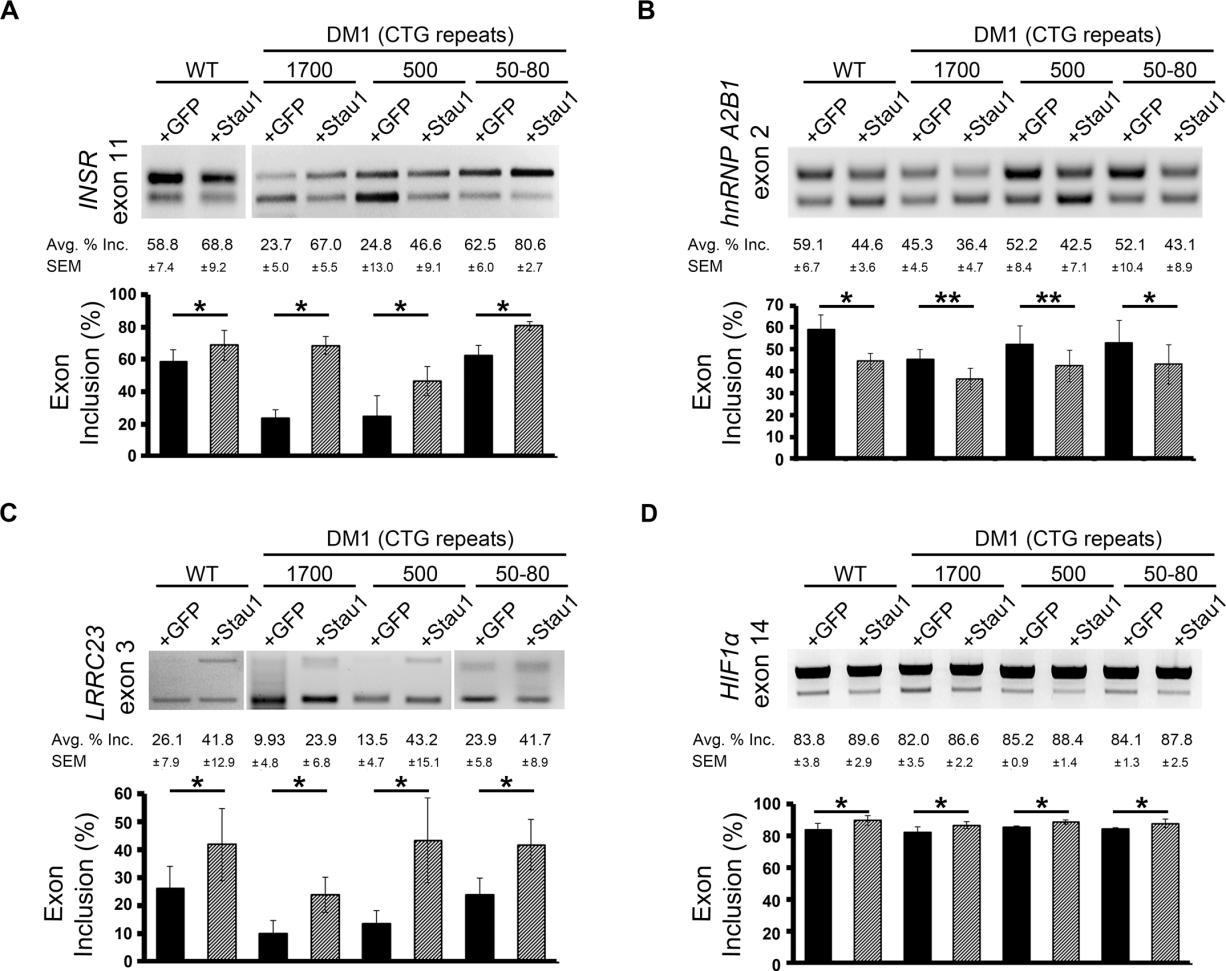
Validation of Stau1-regulated ASEs from RT-PCR splicing screen, in WT and DM1 cell lines. Total RNA was collected from four GM0 cell lines; WT (GM01653) and DM1 cell lines GM03132 (1700 CTG), GM03987 (500 CTG), and GM03991 (50–80 CTG). RT-PCR was performed to determine splicing ratios of ASE in the (A) *INSR* (B) *hnRNPA2B1*, (C) *LRRC23*, (D) *HIF1α* mRNA isoforms. ASE is indicated by exon number for each event. Bar graphs show an average of three independent experiments. Error bars represent SEM * = p < 0.5, ** = p < 0.01.

Of the ASEs affected by Stau1 over-expression in the high-throughput screen, we validated by semi-quantitative RT-PCR 13 of 19 (68%). Among the 13 ASEs that were identified as Stau1-regulated events by our screen and independently shown to be regulated by Stau1 by semi-quantitative RT-PCR validation, we found *INSR*, *hnRNP A2B1*, *LRRC23*, *HIF1α*, *NRG1*, *FN1*, *ACCN3*, *FHL3*, *G6PC3*, *CLCN2* and *CLCN6* (Fig 5A–5D and S3A–S3G Fig). Splicing analysis of four ASEs were extended to include two additional DM1 myogenic cell lines with varying numbers of CTG repeats; 500 CTGs and 50–80 CTGs (Fig 5A–5D and S2B Fig and S2D Fig). These additional DM1 cell lines were included to investigate the influence of Stau1-HA, which was overexpressed at relatively equal amounts (S2D Fig), on splicing regulation in varying degrees of the DM1 pathology. As expected, exon 11 inclusion of the *INSR* decreased with an increase of CTG repeats, and in all cases, Stau1-HA overexpression increased exon 11 inclusion by ≥15%, independent of the number of CTG repeats (Fig 5A). Taken together, the data suggest that Stau1 regulates the splicing of numerous ASEs even in cases of varying degrees of severity of the DM1 pathology.

### Stau1-regulated ASEs contain Alu elements in their flanking introns

**Fig 6:**
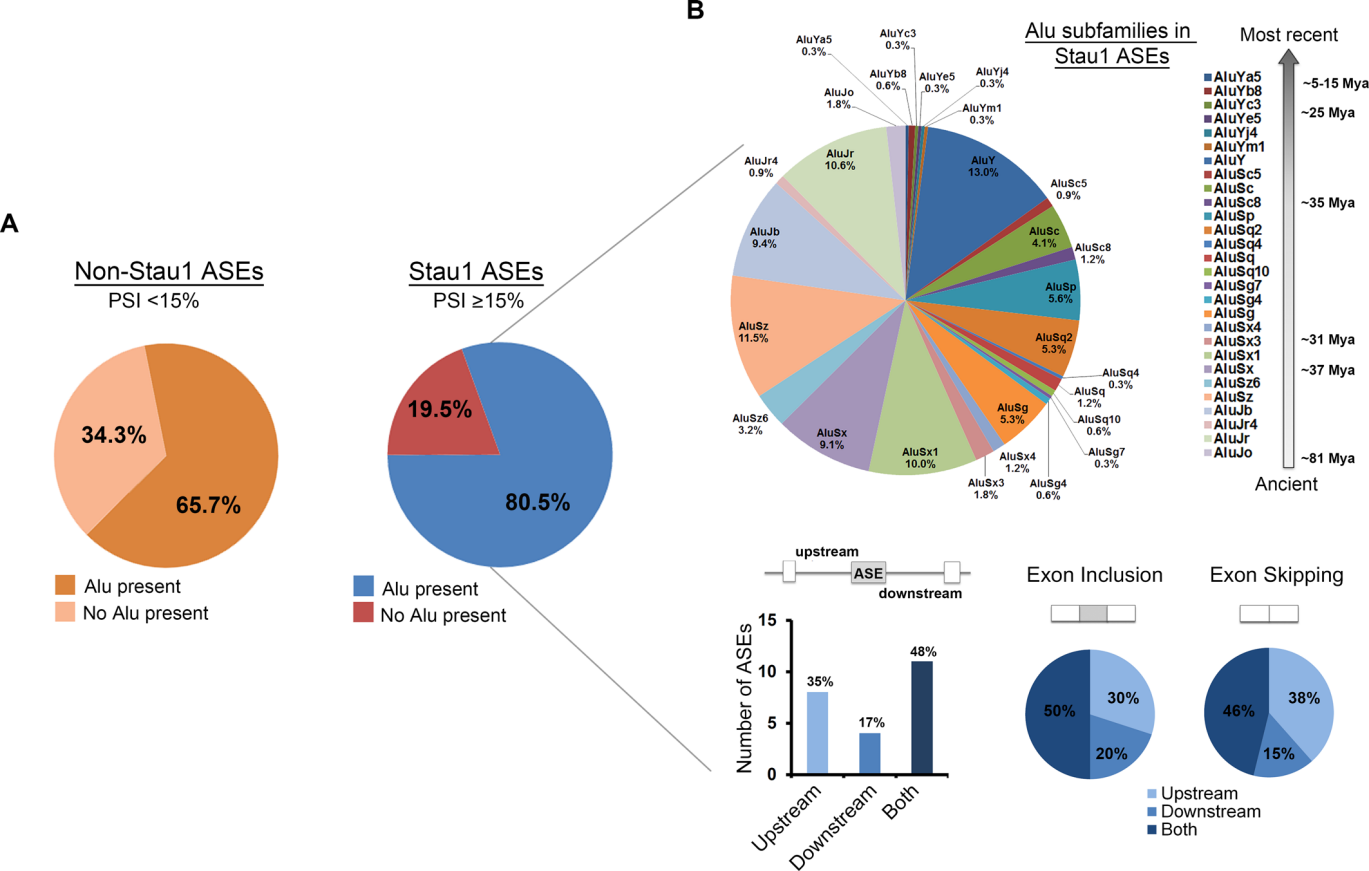
Alu elements in Stau1 and non-Stau1 regulated ASEs. (A) All ASEs were considered for this analysis with the exception of any that had a blank PSI, as described above. Stau1 (≥15% PSI) and non-Stau1 regulated ASEs targets were searched using RepeatMasker software v 4.0.5, to identify the presence of Alu elements in introns flanking the ASE. (B) The subfamilies of Alu elements in Stau1-regulated targets are presented. The locations of Alu elements in Stau1-regulated cassette-type ASE containing targets are represented in a bar graph as the number of Alu elements found in the upstream, downstream or in both introns surrounding the ASE. The locations of these intronic Alu elements were then correlated to the type of splicing event induced by Stau1 overexpression, i.e. exon inclusion or exon skipping.

Given that Alu elements in the upstream intron flanking the alternative exon 11 of the *INSR* is required for both binding and splicing regulation by Stau1 (Fig 2), we examined how many other Stau1-regulated ASEs contained one or more Alu elements as compared to non-Stau1 regulated ASEs. Briefly, we first mapped out the genomic regions corresponding with each primer pairs used in the RT-PCR splicing screen using UCSC Genome Browser (human genomic data v.37). Next, utilizing RepeatMasker (release v.4.0.6), the number of Alu elements that were present within each genomic region of the ASEs was determined. We then compared the number of Stau1-regulated ASEs that harboured Alu elements to non-Stau1-regulated ASE targets. In total, 80.5% of Stau1-regulated targets (ΔPSI ≥15%) contained Alu elements. In contrast, only 65.7% of non-Stau1 regulated targets contained Alu elements (Fig 6A). A similar value of 68.3% was obtained when considering the whole dataset. Focusing on the Stau1 ASEs that contained Alu elements, we again used RepeatMasker to identify the subfamilies of the Alu elements in order to see whether any particular Alu subfamilies were prevalent in Stau1 ASE targets. This analysis revealed no obvious preference toward a specific subfamily of Alu element in the introns flanking Stau1 ASEs (Fig 6B). Further analysis comparing the proportions of the major subfamilies, i.e. AluY, AluS, and AluJ, identified in our study revealed a similar distribution of Alu family proportions to those reported in primate genomes [48, 49]. It has been observed for a number of splicing factors that their recruitment either upstream or downstream of the alternatively spliced cassette-exon correlated with whether they were promoting inclusion or skipping of that exon [21, 50]. To determine if this seemed to be the case for Stau1, we examined the distribution of Alu elements relative to the alternative cassette-exon amongst top Stau1-regulated ASEs. This analysis revealed that the 35% of Alu elements were found within the upstream introns, relative to 17% in downstream introns, while the remaining 48% were found in both flanking introns (Fig 6B). However, this distribution did not seem to correlate strongly with whether Stau1 induced exon inclusion or skipping for those ASEs.

Since Stau1 has also been proposed to bind to various non-Alu RNA secondary structures [35, 37], the predicted secondary structures of the flanking introns of three non-Alu containing Stau1-regulated ASEs, were scanned for RNA secondary structures that resembled possible SBS. This additional analysis revealed the presence of secondary structures resembling known or predicted SBS within the proximity of the ASEs of *hnRNP A2B1*, *LRRC23*, and *NRG1* (S4A–S4C Fig). These may serve as SBS allowing Stau1 to regulate the splicing of these pre-mRNAs when no Alu elements are present.

## Discussion

We report here that Stau1 regulates the alternative splicing of *INSR* exon 11 through its interaction with Alu elements located in intron 10. Using a high-throughput alternative splicing screen, we also demonstrate that Stau1 regulates a broad range of pre-mRNAs, many of which also harbour Alu elements within introns flanking the ASE. Importantly, although Stau1 overexpression in DM1 myoblasts did rescue splicing patterns of many pre-mRNAs towards WT, as previously observed for *INSR* exon 11, a number of Stau1-induced splicing changes were also found shifting away from WT patterns, and thus would be predicted to exacerbate the DM1 pathology. Taken together, these findings are consistent with the notion that Stau1 may act as a disease modifier in DM1.

### Stau1 regulates alternative splicing of *INSR* exon 11 through binding to Alu elements

In the present study, we demonstrated that modulation of Stau1 levels regulates *INSR* exon 11 splicing. This regulation was demonstrated in two non-muscle (HeLa and HEK293Ts) cell lines and complements our previous findings in DM1 muscle cell lines [32]. Modulating Stau1 levels did not result in major changes in the expression of other splicing factors known to regulate exon 11 inclusion, such as MBNL1, CUGBP1 or hnRNP H. Furthermore, our previous work found no protein-protein interactions between Stau1 and MBNL1 or CUGBP1 [32]. Taken together, these findings support the idea that Stau1 regulates splicing without direct protein interactions or modulation of the expression levels of these other key splicing factors. Nevertheless, Stau1 may still affect the functional activity of these splicing factors in ways that are more indirect.

**Fig 7:**
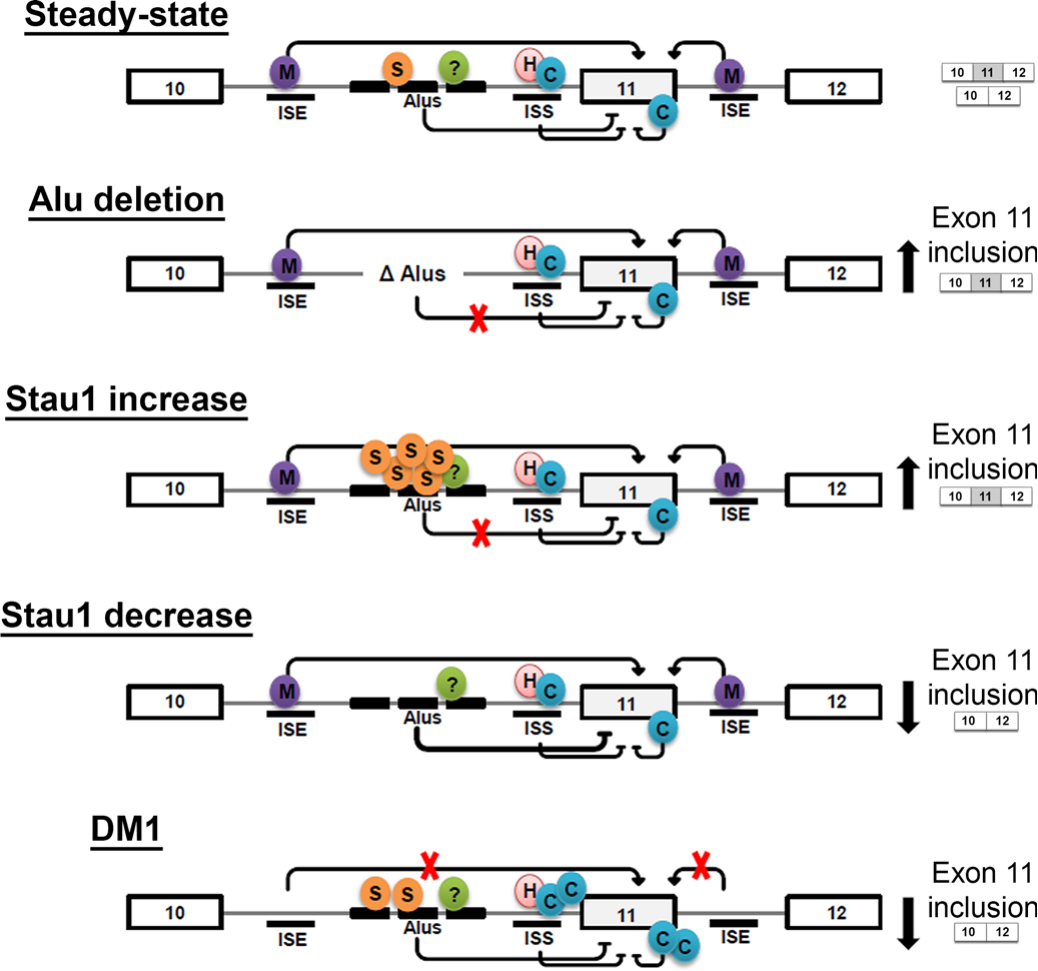
Proposed model of how Stau1 may contribute to *INSR* exon 11 alternative splicing. At steady-state, Stau1’s binding to Alu elements participates in the integration of several regulatory mechanisms towards establishing the level of utilisation of exon 11. Introns are denoted as grey lines and exons as large boxes. The splicing factors MBNL1 (M), CUGBP1 (C), hnRNP H (H), and Stau1 (S) are depicted binding to various known cis-regulatory elements (i.e. intronic splicing enhancer (ISE) and intronic splicing silencers (ISS)) and to the Alu elements (Alus). Complete deletion of the Alu elements results in increased exon 11 inclusion, suggesting they normally contribute towards a repressive influence on exon inclusion. Upon overexpression of Stau1, increased exon 11 inclusion is also observed, suggesting Stau1 somehow disrupts the repressive influence of the Alu elements (see Discussion for details). Upon knockdown of Stau1 expression, the full inhibitory effect of the Alu elements would be observed. In DM1, the combined effects of reduced MBNL1 levels increased CUGBP1 activity, and a moderate increase in Stau1 would result in decreased exon 11 inclusion.

Alu elements serving as cis-regulatory elements for splicing have been described. For example in the *RABL5* pre-mRNA, two Alu elements that were in opposite orientation in the upstream intron were shown to affect the splicing patterns of the downstream exon 3 [51]. Another example is the regulation of the alternative splicing in the Ataxia Telangiectasia Mutated (*ATM*) pre-mRNA. In this case, an intronic splicing element derived from an Alu element was found to modulate the inclusion of a cryptic exon [52]. In a number of cases, binding of trans-acting factors to Alu elements is required to mediate an effect on splicing [46, 53]. For example, Zarnack and colleagues demonstrated that, in the absence of hnRNP C, thousands of Alu elements were included as exons in mRNA transcripts [53]. Splicing analysis of IR-minigenes in HeLa cells demonstrated a drastic shift in the splicing pattern to exon 11 inclusion occurred when the Alu elements in intron 10 were deleted. These findings agree with previous literature reporting similar results in HepG2 cells [47]. Thus, we propose that the presence of Alu elements serves to inhibit the inclusion of exon 11, perhaps through the recruitment of one or more Alu-element binding trans-acting factor(s), in addition to Stau1, that would then interfere with recruitment of constitutive splicing factors (see model in Fig 7). It may be informative to examine any potential interactions between Stau1 and known Alu-binding splicing proteins to further define the mechanism by which Stau1 regulates alternative splicing.

Our results show that increased Stau1 levels correlate with increased inclusion of exon 11. The specific details of what occurs once more Stau1 binds to the Alu elements remains unclear. As depicted in Fig 7, this could somehow prevent the inhibitory effect of the Alu repeats on exon 11 inclusion, either by preventing recruitment of Alu binding factors or through recruitment of a distinct factor that would instead favour inclusion. Conversely, upon Stau1 knock down, this would allow for the full inhibitory effect of the Alu elements on exon 11 inclusion (Fig 7). A number of additional factors have been reported to regulate alternative splicing of exon 11 (e.g. hnRNP H, DDX5, etc) [54] and we cannot rule out a mechanism whereby Stau1 binding to the Alu elements may modulate the action of one or more of these factors. Finally, in the context of DM1, where we know the positive contribution of MBLN1 is lost and the inhibitory effects of CUGBP1 are enhanced, the moderate increase in Stau1 levels that we have documented in patient tissues, may not be enough to completely prevent the inhibitory contribution of the Alu elements on exon 11 inclusion (Fig 7). Alternatively, Stau1’s activity might be misregulated in DM1 (e.g. through post-translational modification(s) and/or interaction with distinct factors). Further experimentation will be required to determine the precise mechanism(s) by which Stau1 contributes to *INSR* splicing under both normal and DM1 settings. Nevertheless, to our knowledge, this is the first example showing that the binding of Stau1 to Alu elements can regulate alternative splicing.

### Stau1 can affect the alternative splicing of a broad range of pre-mRNAs

Strikingly, our high-throughput screen has revealed that Stau1 can regulate alternative splicing to an extent that is comparable to well-established splicing regulators like MBNL1 and RBFOX1 (see Fig 4D). An analysis of all ASEs examined in this study revealed that Stau1-regulated ASEs were more likely to harbour one or more Alu elements than those that were not Stau1-sensitive (80.5% vs. 65.7%, respectively). Amongst Stau1-regulated pre-mRNAs Alu elements were found within both introns flanking the ASE for ~50% of cases. Recent studies that investigated SBS uncovered that Stau1 preferentially binds complex, imperfectly paired duplex structures formed by the pairing of at least two Alu elements [37, 55]. Thus, it would be interesting in future work to investigate whether Alu elements found on either side of an ASE need to form duplexes in order to influence splicing decisions. Genome-wide occupancy assessment of splicing regulators, using, for example, HITS-CLIP or similar approaches, has revealed that binding of splicing regulators either upstream or downstream of an ASE is often correlated with whether it will mediate a positive or negative effect on that splicing event [50]. Using a limited dataset of ASEs most significantly affected by Stau1, we determined that when Alu elements (and thus potential SBSs) were not present in both flanking introns, they were most often found in introns upstream of the ASE (~twice as often than in downstream intron; see Fig 6). However, we found no correlation between Alu elements being present upstream or downstream of ASEs and whether the effect of Stau1 on that ASE was the induction of skipping or inclusion. We did not document within this limited dataset Alu elements positioned in very close proximity or overlapping with splice sites, but this is obviously another aspect that would need to be considered. These questions will require analysis of larger datasets in order for more conclusive patterns to emerge.

Although a majority of Stau1 ASEs harboured Alu elements in introns flanking the ASE, we observed that ~20% ASEs affected by Stau1 level modulation did not contain any Alu elements. Further analysis into the RNA secondary structure of non-Alu containing introns flanking ASEs revealed the presence of RNA duplex structures, which may represent potential SBSs. Interestingly, the three potential non-Alu SBSs in the Stau1 targets *hnRNPA2B1*, *NRG1* and *LRRC23* (S4 Fig) are all located close to the 3’SS, which could thus interfere with recognition of this site by the basic splicing machinery; a phenomenon that Stau1 could then either promote or interfere with. Several pre-mRNAs containing ASEs that were sensitive to Stau1 level modulation have been previously identified as Stau1 targets. Specifically, Stau1 binds to these targets through non-Alu element binding sites located in the coding sequence of the transcripts, including: *ADPGK*, *AKT2*, *ANK1*, *APOBEC3C*, *ARF1*, *ARFRP1*, *ENSA*, *FN1*, *JAG2*, *NUTF2*, *OGDH*, *SHKBP1*, *SORBP1*, *TBC1D12/13*, and *THRA* [31, 36, 37].

### Stau1 as a disease modifier in DM1

Over the past decades, the major emphasis in defining the molecular pathogenesis of DM1 has focused on the role of a very few specific RNA-binding proteins, such as MBNL1 and CUGBP1, in aberrant alternative splicing events in DM1. Although animal models of MBNL1 and CUGBP1 [56, 57] do reproduce many DM1 symptoms [43, 58], some are not recapitulated, suggesting that other factors, such as disease modifiers are involved. Indeed, several disease modifier proteins have recently been identified and shown to have an impact on the DM1 pathology. For example, in a Drosophila DM1 model the RNA-binding proteins TBPH (homolog of human TAR DNA-binding protein 43 or TDP-43) and BSF (Bicoid stability factor; homolog of human LRPPRC) were found be misregulated with the expression of CUG expansions resulting in altered muscle sarcomere location of these proteins [59]. Another study, done by Huin and colleagues, reported that several genetic variants of the *MBNL1* gene could be associated with the severity of the disease, suggesting that these variants were acting as disease modifiers in DM1. [60]. Finally, the DEAD-box RNA helicase, DDX5/p68, found to be reduced in DM1 biopsied skeletal muscle [61], was shown to allow increased MBNL1 binding to mutant repeats which can influence splicing events misregulated in DM1 [62].

In agreement with our initial study [32], the data presented here show that overexpression of Stau1 resulted in several splicing events predicted to be beneficial for DM1, such as the rescue of the *INSR* exon 11. However, we also identified a number of detrimental splicing effects, which would likely exacerbate the DM1 pathology (see Fig 4A and 4B). This suggests that the upregulation of Stau1 may not represent a protective role in the DM1 pathology as previously suggested but, instead, shows Stau1 likely acts as a disease modifier for DM1 whose splicing impact can result in both beneficial and detrimental effects on the DM1 phenotype. Additionally, it is possible that Stau1 may act as a disease modifier in DM1 through non-splicing related activities. For example, we have recently shown that Stau1 negatively regulates skeletal muscle differentiation, at least in part through its regulation of c-myc translation [63]. As such, Stau1 may thus contribute to the impaired differentiation/developmental program observed in DM1 [64]. The precise overall contribution of Stau1 to the DM1 phenotype thus remains to be fully explored, but our results to date strongly indicate that Stau1 needs to be considered amongst the gene products that modulate the complex DM1 pathophysiology and its response to future therapeutic interventions.

## Materials and Methods

### Cell lines

The following cell lines were obtained from the NIGMS Human Genetic Cell Repository at the Coriell Institute for Medical Research: WT cell lines were represented by two cell lines with 0–5 CTGs repeats: GM03377 (Splicing screen) and GM01653 (Validation). DM1 cell lines used were GM03132 (1700 CTGs), GM03987 (500 CTGs) and GM03991 (50–80 CTGs). HeLa cell lines were obtained from ATCC (ATCC CCL-2) and HEK293-T cell lines were obtained from ATCC (ATCC CRL-1573).

### Plasmid constructs, cell transfections and infections and cell lysis

Constructs: IR-minigenes WT and ΔAlus (aka IR-B and IR-E) were generously donated by Nicholas Webster and previously described in [47], MyoD virus (pBRIT-MyoD-(His-TEV-3FLAG)), GFP virus, hStau1^5^-HA plasmid (described in [24]), shStau1 plasmid mix made up of two shRNAs against human Stau1 mRNA (referred to in text as shStau1) (Open Biosystems GE Dharmacon: sh06 [Clone ID:TRCN0000102306] + sh09 [Clone ID:TRCN0000102309]). Cells to be transfected were grown to ~70% confluency and transfected with 1–3 μg of DNA using Lipofectamine with plus reagent (Life Technologies:15338100), according to manufacturers protocol, for 48 hours. Virus production consisted of using plasmids previously described [32] pcDH-CMV-MCS-EF1-copGFP and pcDH-Stau1. Viral particles were produced by transient transfection of HEK-293T cells with lentiviral packaging vectors psPAX2 (Addgene:12260) and pMD2.G (Addgene:12259) using Lipofectamine 2000 reagent (Life Technologies:11668027) according to manufactures protocol. The conditioned medium containing viral particles was collected and used to transduce control and DM1 myoblasts overnight in the presence of 8ug/ml Hexadimethrine Bromide (SIGMA:H9268). Subsequent infections were performed the following day, and cells were grown for several days before analyses. Infection of cells involved growing cells until ~70% confluency, infecting first with MyoD virus, selection with Puromycin (Wisent Bio Products:400-160-EM) (1μg/mL) for 5 days, infection with either the GFP or hStau1^55^-HA virus, confirming GFP expression after 48 and harvesting cells for RNA and protein 72 hours after initial second infection. Cells were washed with 1XPBS, scraped and lysed in 1 mL of RIPA buffer with Protease Inhibitor added prior to use. Cells with incubated in RIPA buffer for 30 min on ice and centrifuged for 15 minutes at 13,200 rpm. The supernatant was collected and stored at -20°C until use.

### Western blotting analysis and antibodies

Following cell lysis, protein concentration was assessed using the Bio-Rad DC Protein Assay (Bio-Rad:500–0111) and protein (2–40 μg) was resolved by denaturing polyacrylamide gel electrophoresis (SDS-PAGE) and transferred to polyvinylidene fluoride (PDVF) membranes (Immunobilon Transfer Membranes:IPVH00010). Transferred membranes were blocked with 5% milk for 30 minutes and probed with appropriate antibody in 1% milk solution for either 1 hour at room temperature or 12 hours at 4°C, with three 10 min washes with 1XPBS-0.05% Tween 20 between each antibody incubation. Antibodies included: Anti-Stau1 [1:1000] (Abcam:ab73478), Anti-GAPDH [1:10,000] (Abcam:ab8245), Anti-β-Actin [1:500] (Santa Cruz:sc-47778), Anti-CUGBP1 [1:1000] (Santa Cruz:sc-20003), Anti-hnRNP H [1:5000] (Abcam:10374), Anti-MyoD [1:300] (BD Pharmingen: 554130), Anti-MBNL1 antibody [1:300] (Abnova:H00004154), Anti-HA F7 probe [1:1000] (Santa Cruz:sc-7392). Secondary antibodies included: Mouse-anti-Rabbit HRP [1:20,000] (Jackson ImmunoResearch:211-032-171) and Goat-anti-Rabbit HRP [1:10,000] (Molecular Probes:MP 02764). Proteins on membranes were detected with Millipore-Luminata Crescendo Western HRP Substrate (WBLUR0500) and visualized on film (HyBlot CL Autoradiography Film:E3018).

### RNA extraction and cDNA synthesis

RNA was isolated from whole cell lysates using Ambion TRIzol Reagent (E3018) and 2μL of collected RNA was assessed on the Take3BioPlate Reader to determine quantity (ng/μL) and quality (RNA with 260/280 ~ 2.0 was used). 500 ng of RNA was used to synthesize cDNA with random hexamers (10mM) and the Promega AMV cDNA synthesis (Promega:M5101) was carried out following manufacturer's protocol. cDNA was diluted 1:20 and 5μL (~100 ng) was used for each RT-PCR and RT-qPCR reaction. All cDNA and RNA was stored at -20°C short-term and -80°C long term.

### Reverse Transcription (RT)- PCR and quantitative RT- PCR (RT-qPCR)

RT-PCR was performed using Promega GoTaq DNA Polymerase (Promega:M5101) according to manufacturer's protocol. RT-PCR conditions were as follows for validation of splicing screen: 95°C for 2 min, (95°C for 30 sec, 55°C for 30 sec, 72°C for 45 sec)x32 cycles, 72°C for 10 min. Specific RT-PCR conditions used for particular primers are available upon request. Amplicons were run on a 2% agarose gel (containing 3–5 μL of EtBr 20 mg/mL) and visualized under UV light. All RT-qPCR reactions were performed using BioRad iQ SYBR Green Supermix (BioRad:170–8882) according to manufactures protocol and run with a Chromo 4 Real-Time PCR Detector. RT-qPCR conditions for all primer sets used were carried out as follows: 95°C for 2 min, (95°C for 30 sec, 60°C for 30 sec, 72°C for 45 sec)x40 cycles, 72°C for 10 min. Technical replicates of 3 were done for all RT-qPCR experiments and the average Ct values were normalized to either *GAPDH* or 18S (indicated in descriptions). The ΔΔCt method was used to analyze fold change of transcripts. Primers for *INSR* splicing analysis were previously described [32], and any of the 487 primer sets used in our RT-PCR splicing screen are available upon request. Biological replicates of ≥3 samples were done for all PCR reactions.

### High-throughput RT-PCR splicing screen

RNA from (WT) GM03377 and DM1-1700 CTG (GM03132) was synthesized to cDNA and subject to the screen as previously described [18]. Raw data from the screen is included in S1 Table. Analysis of data to determine top ASE included rankings of events that had the greatest PSI change (Δ) between two conditions, i.e. the ΔPSI between WT+GFP virus and WT+Stau1 virus:

$$\Delta \text{PSI}={\text{PSI}}^{\text{GFP}}{\text{-PSI}}^{\mathrm{Stau}1\text{-HA}}$$ (1)

Only values with a ΔPSI ≥10% between conditions were selected for additional analysis:

$$\left[\left(\text{PSI}{\left(\text{WT}\right)}^{\text{GFP}}\text{-PSI}{\left(\text{DM}1\right)}^{\text{GFP}}\right)\left({\sqrt{\text{-}\left(\text{n}\right)}}^{2}\right)\right]*100\geq 10\%$$ (2)

ASEs which contained PSI values with no data (no isoforms detected), were not included in analyses.

### Validation of ASEs regulated by Stau1

A minimum of three biological replicates were used to validate a change in the splicing patterns (ΔPSI) for all cell lines tested. Semi-quantitative RT-PCR was carried out with the required primers to obtain two isoforms describing an ASE for the splicing screen. Splicing patterns in each condition (WT+GFP, WT+Stau1-HA, DM1+GFP, DM1+Stau1-HA) was analyzed and classified as successfully tested and validated if a change in splicing pattern was detected in all three biological replicates and followed the same splicing pattern as predicted by our screen for all replicates (n = 3). ASEs were classified as successfully tested but not validated if the splicing pattern detected in all three biological replicates did not change between conditions or did not follow the splicing pattern predicted by the screen.

### RNA Immunoprecipitations (RIPs)

48 hours after transfection cells were treated with 1% formaldehyde to induce cross-link *in vivo* Stau1-HA-RNA complexes for 10 minutes at RT and reaction was quenched with 0.25 M glycine in PBS. Cells were suspended in 1 mL of RNase-free RIPA buffer and centrifuged for 15 minutes at 13, 200 rpm at 4°C. The supernatant was collected and centrifuged twice more. 40 μL of Santa Cruz Protein A/G PLUS agarose beads (Santa Cruz:sc-2003) suspended in RNase-free RIPA buffer was added to lysate was incubated with gentle rotation for 1 hour at 4°C to pre-clear. Beads were removed by centrifugation and the pre-cleared lysate was aliquoted by volume into 10% input, IgG and IP. Normal mouse IgG antibody (Santa Cruz:sc-2025) or mouse-anti HA antibody (4 μg) was added to IgG or IP, respectively, and incubated for 16 hours at 4°C with gentle rotation. 40 μL of A/G plus beads were added to IgG and IP samples and incubated for 1 hour at 4°C with gentle rotation. Stau1-RNA complexes bound to beads were pelleted at 2,500 rpm for 30 sec, supernatant removed and the pellet was resuspended in 1 mL of RNase-free RIPA buffer. These washes were repeated three times. Following final resuspension of pellet, crosslinking was reversed (1 hour at 70°C). Trizol was then directly added to the bead-RIPA solution and RNA isolation protocol was followed (as described above).

### Identification of SBS (Alu elements and potential non-Alu elements) flanking ASEs

Primer pairs in the high-throughput RT-PCR splicing screen were used in a BLAST to identify the mRNA transcript ASE that Stau1 was suspected to regulate. Using CLC MainWorkbench, alignments of the DNA and mRNA transcripts revealed the exon(s) and flanking introns, which defined the ASE. Flanking introns were then analyzed with RepeatMasker (v.4.0.6) to identify the presence and subfamily of Alu element(s). Exons that made up the ASE were also analyzed for the presence of Alu elements however, none was found at that time. A total of 23 cassette exon type Alu element containing Stau1-regulated ASE targets were examined to identify the location of the Alu(s), either upstream or downstream of the ASE. These were then categorized by whether Stau1 overexpression induced exon inclusion or skipping of the cassette exon. If an intronic sequence did not contain any Alu elements, the MFE structure of the intronic sequences were manually searched for potential duplexes resembling SBS. These potential SBS were identified based on previous reports describing identified SBS, [36, 37] i.e. average size of stem length (duplex) (between 5–19 base pairs), high base pair probability and varying degrees of imperfect base pairing (preference given to longest continuous duplex structure formation). If the intronic sequence was >7,500 bps (current limit of RNAfold partition function calculations), then intron was divided into equal segments, each ≤7,500 bps, and subsequent predicted RNA secondary structure was used.

### Statistical analysis

To identify statistical significance between two groups one-tailed student's t-tests were carried out on data with biological replicates n≥3. The level to determine a value as significant was set as p<0.05. Significance was denoted as follows: * p<0.05, ** p<0.01, ***p<0.001.

## Supporting Information

S1 Fig Stau1 levels regulate the pre-mRNA splicing of the human *INSR* in HEK293Ts cells. (A) pGIPZ (CTRL), shStau1 or Stau1-HA (Stau1-HA) plasmids were transiently transfected into HEK293T cell lines and total RNA and protein lysate was collected after 48 hours. RT-PCR using primers specific to the human endogenous *INSR* were used on cDNA synthesized from total RNA to amplify the two isoforms (IR-A and IR-B) of the *INSR*. (B) Stau1, CUGBP1, MBNL1 and hnRNP H protein levels were assessed by Western blot using β-actin or GAPDH as a loading control. (C) Semi-quantitative RT-PCR using primers specific to the human Stau1 mRNA demonstrates the increase and decrease of Stau1 mRNA in HeLa cell lines. RT-qPCR using primers specific to the human *MBNL1*, *CUGBP1*, and *hnRNP H* mRNA transcripts in HeLa cell lines with decreased or overexpressed Stau1 levels. 18S was used as for normalization in PCR experiments. In all cases, bar graphs show an average of ≥3 independent experiments. Error bars represent SEM * = p < 0.05, ** = p < 0.01, *** = p < 0.001.
(TIF)

S2 Fig Confirmation of MyoD expression and overexpression of Stau1-HA protein in GM0 cells. (A) Representative image of GFP positive MyoD converted WT and DM1 cell lines. (B) One WT (GM03377) and three DM1 (GM03132, GM03987, GM03991) primary fibroblast cell lines were converted to myoblasts using MyoD retrovirus. Semi-quantitative RT-PCR using primers specific to amplify *MyoD* plasmid demonstrates plasmid expression in all MyoD converted myoblast cell lines as compared to uninfected fibroblast cell lines. 18S was used as a loading control. (C) Protein was collected from GM03132 cell lines and western blot was used to analyze the levels of MyoD protein from virus infected MyoD converted myoblasts compared to uninfected fibroblast cell lines. β-actin was used as a loading control. (D) Representative Western blot showing levels of Stau1-HA in MyoD converted myoblast GM0 cell lines as compared to GFP infected MyoD converted cell lines. β-actin was used as a loading control.
(TIF)

S3 Fig Additional validation of high-throughput RT-PCR splicing screen. (A-G) Total RNA was collected from WT and DM1 (1700 CTG) cell lines. Semi-quantitative RT-PCR was performed to determine splicing ratios of (A) *NRG1*, (B) *FN1*, (C) *ACCN3*, (D) *FHL3*, (E) *G6PC3*, (F) *CLCN2* and (G) *CLCN6* mRNA long and short isoforms. ASE is indicated by exon number for each event. Bar graphs show an average of three independent experiments. Error bars represent SEM * = p < 0.05, ** = p < 0.01, *** = p < 0.001.
(TIF)

S4 Fig Proposed SBS in validated Stau1-regulated ASEs. The genomic DNA sequence of the human (A) *hnRNPA2B1* (NG_000007.14) (B) *NRG1* (NG_000008.11) and (C) *LRRC23* (NG_000012.12) was used to assess the possible non-Alu SBS. RNA secondary structure of indicated introns was determined by Vienna package RNAfold 2.1.1 and identification of possible SBS were determined following guidelines described in the materials and methods.
(TIF)

S1 Table RT-PCR splicing screen information. This excel file contains: Tab 1: The raw data PSI values from all four conditions performed in this study, Tab 2: The ASEs PSI values from DM1 conditions compared to CTRL from Klinck et al., 2014, Tab 3: The comparison between ASEs in DM1 between Klinck et al., 2014 and the splicing screen from the current study, Tabs 5 and 6: Comparison between ASEs regulated by Stau1 to MBNL1 and/or RBFOX1. All PSI≥5% were considered for any analysis comparing our data with that our Klinck *et al*., 2014., Tab 6: The subfamilies of Alu elements in Stau1-regulated targets.
(XLSX)

S2 Table Analysis of Stau1-regulated ASEs associated with disease. (PDF)
