# Supplementary figures and images for: Staufen1 Regulates Multiple Alternative Splicing Events either Positively or Negatively in DM1 Indicating Its Role as a Disease Modifier
Source: PLoS Genet. 2016 Jan 29;12(1):e1005827. doi: 10.1371/journal.pgen.1005827 (PMC4733145; doi:10.1371/journal.pgen.1005827)

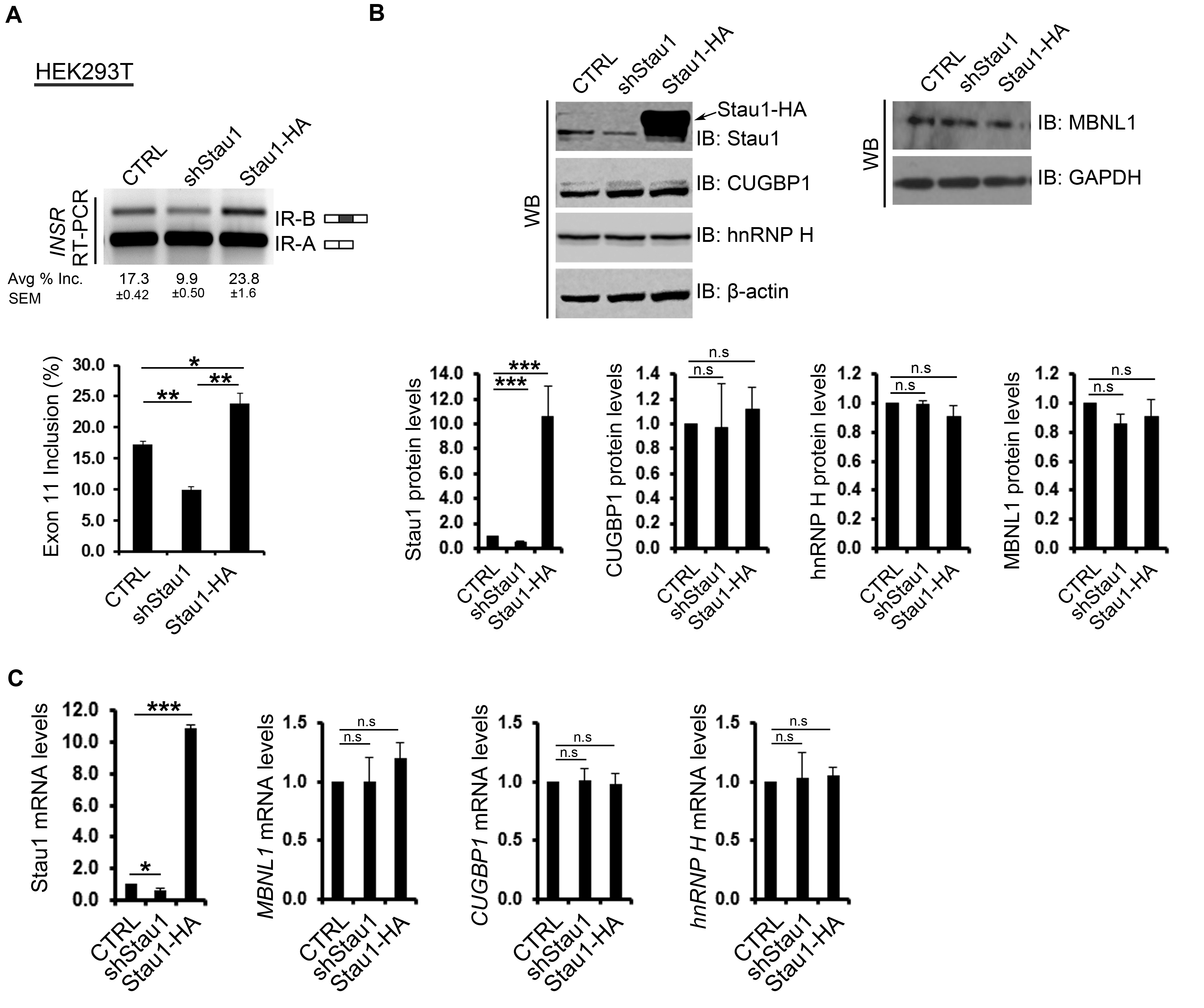

Supplement: S1 Fig — (A) pGIPZ (CTRL), shStau1 or Stau1-HA (Stau1-HA) plasmids were transiently transfected into HEK293T cell lines and total RNA and protein lysate was collected after 48 hours. RT-PCR using primers specific to the human endogenous INSR were used on cDNA synthesized from total RNA to amplify the two isoforms (IR-A and IR-B) of the INSR. (B) Stau1, CUGBP1, MBNL1 and hnRNP H protein levels were assessed by Western blot using β-actin or GAPDH as a loading control. (C) Semi-quantitative RT-PCR using primers specific to the human Stau1 mRNA demonstrates the increase and decrease of Stau1 mRNA in HeLa cell lines. RT-qPCR using primers specific to the human MBNL1, CUGBP1, and hnRNP H mRNA transcripts in HeLa cell lines with decreased or overexpressed Stau1 levels. 18S was used as for normalization in PCR experiments. In all cases, bar graphs show an average of ≥3 independent experiments. Error bars represent SEM * = p < 0.05, ** = p < 0.01, *** = p < 0.001. (TIF) [file pgen.1005827.s001.TIF]

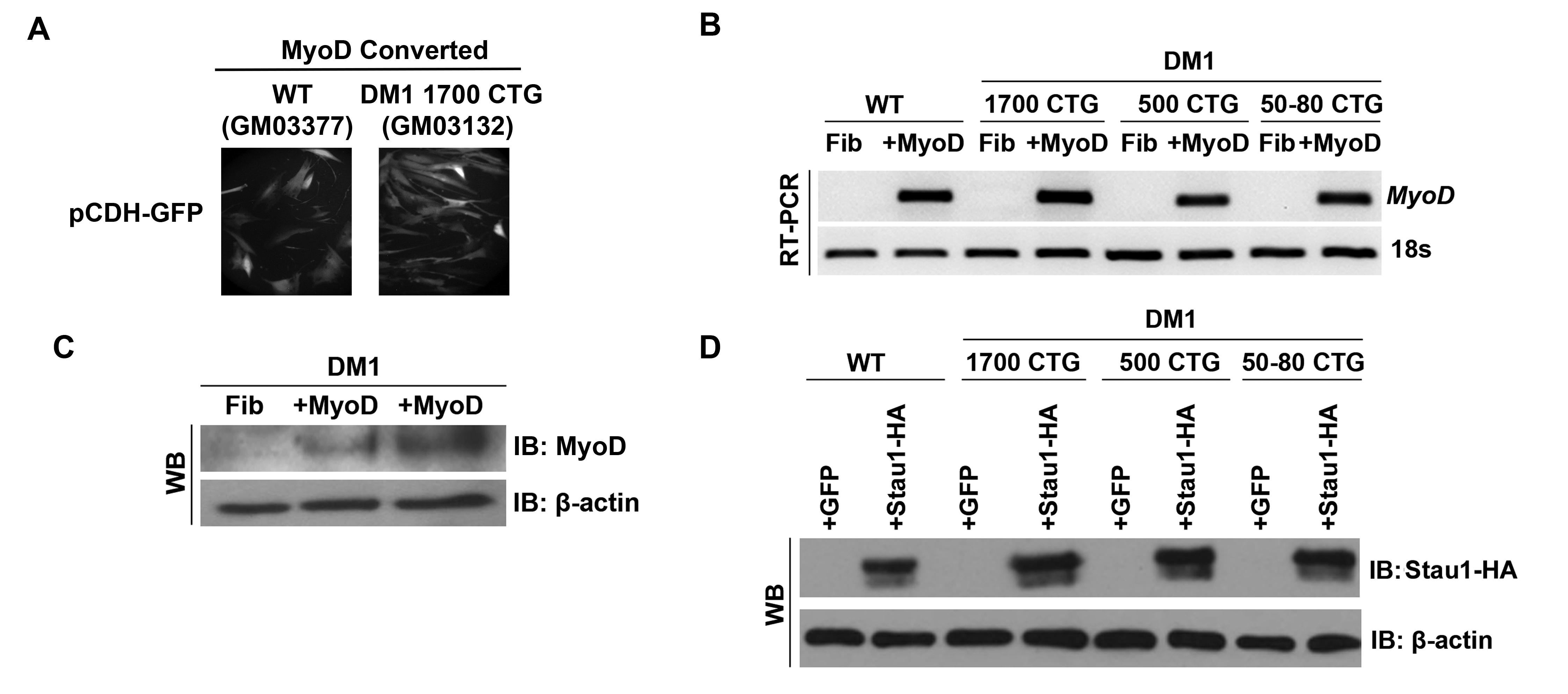

Supplement: S2 Fig — (A) Representative image of GFP positive MyoD converted WT and DM1 cell lines. (B) One WT (GM03377) and three DM1 (GM03132, GM03987, GM03991) primary fibroblast cell lines were converted to myoblasts using MyoD retrovirus. Semi-quantitative RT-PCR using primers specific to amplify MyoD plasmid demonstrates plasmid expression in all MyoD converted myoblast cell lines as compared to uninfected fibroblast cell lines. 18S was used as a loading control. (C) Protein was collected from GM03132 cell lines and western blot was used to analyze the levels of MyoD protein from virus infected MyoD converted myoblasts compared to uninfected fibroblast cell lines. β-actin was used as a loading control. (D) Representative Western blot showing levels of Stau1-HA in MyoD converted myoblast GM0 cell lines as compared to GFP infected MyoD converted cell lines. β-actin was used as a loading control. (TIF) [file pgen.1005827.s002.TIF]

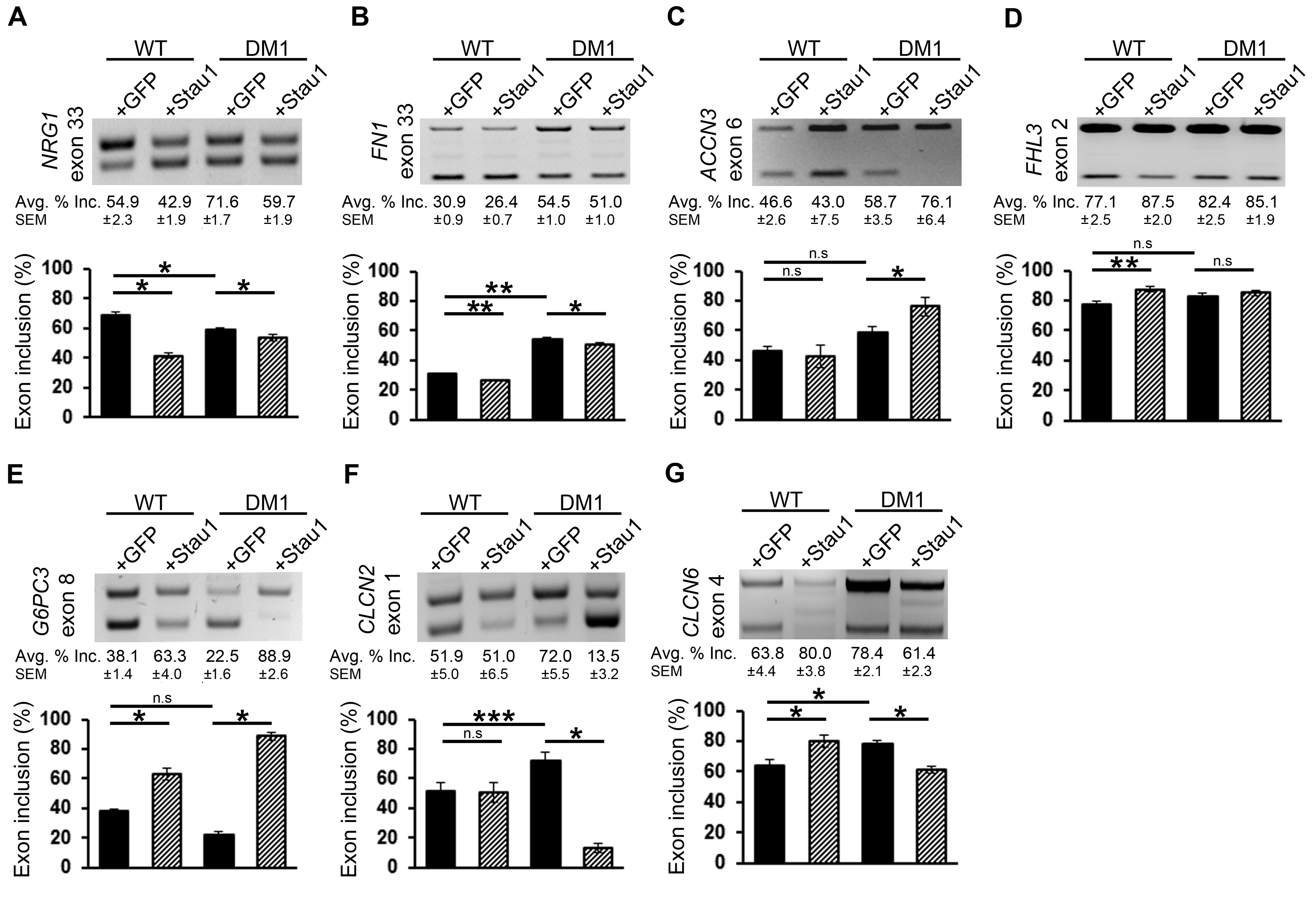

Supplement: S3 Fig — (A-G) Total RNA was collected from WT and DM1 (1700 CTG) cell lines. Semi-quantitative RT-PCR was performed to determine splicing ratios of (A) NRG1, (B) FN1, (C) ACCN3, (D) FHL3, (E) G6PC3, (F) CLCN2 and (G) CLCN6 mRNA long and short isoforms. ASE is indicated by exon number for each event. Bar graphs show an average of three independent experiments. Error bars represent SEM * = p < 0.05, ** = p < 0.01, *** = p < 0.001. (TIF) [file pgen.1005827.s003.TIF]

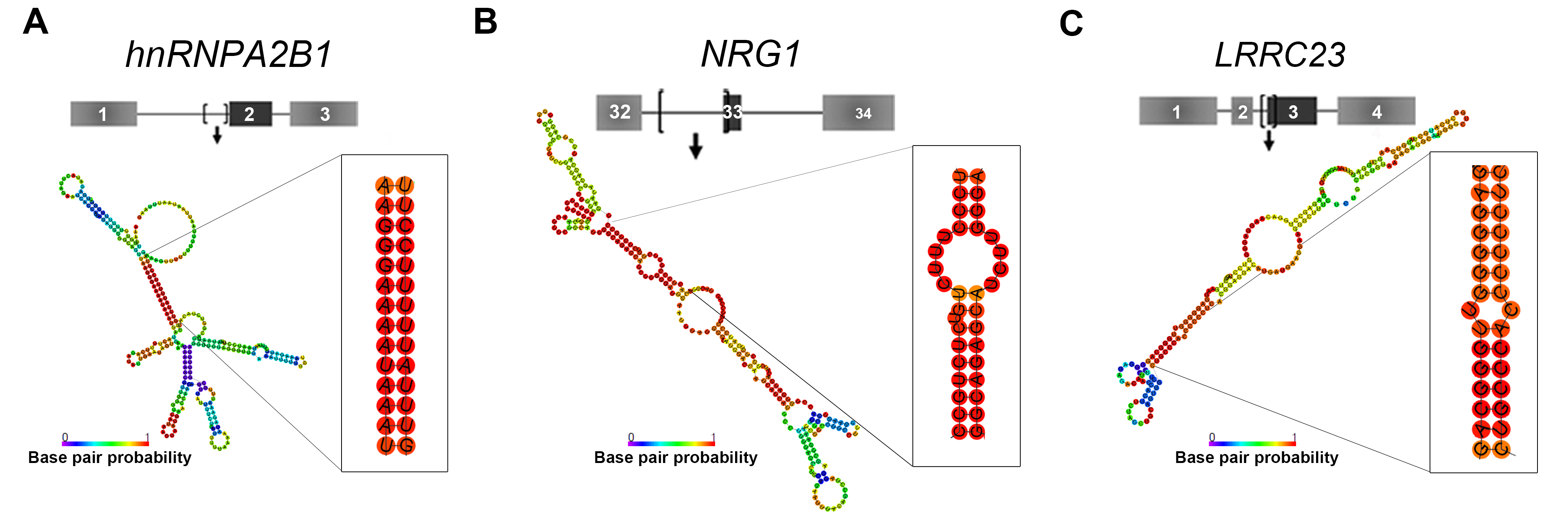

Supplement: S4 Fig — The genomic DNA sequence of the human (A) hnRNPA2B1 (NG_000007.14) (B) NRG1 (NG_000008.11) and (C) LRRC23 (NG_000012.12) was used to assess the possible non-Alu SBS. RNA secondary structure of indicated introns was determined by Vienna package RNAfold 2.1.1 and identification of possible SBS were determined following guidelines described in the materials and methods. (TIF) [file pgen.1005827.s004.TIF]
