# Supplementary material for: Staufen1 Regulates Multiple Alternative Splicing Events either Positively or Negatively in DM1 Indicating Its Role as a Disease Modifier
Source: PLoS Genet. 2016 Jan 29;12(1):e1005827. doi: 10.1371/journal.pgen.1005827 (PMC4733145; doi:10.1371/journal.pgen.1005827)
Supplement: S2 Table — (PDF) [file pgen.1005827.s006.pdf]

S2 Table. Validation Genes and Disease

Gene symbol	Gene name	ASE	Stau1 OE WT	Stau1 OE DM1	Predicted by screen	Variant previously reported	Reported variant name (Δexon)	Splice variant disease related?
INSR	Insulin Receptor	Exon 11	Inclusion	Inclusion	Y	Y - Savkur et al., 2001	IR-A	Y – DM1 - Savkur et al., 2001
hnRNP A2B1	Heterogeneous nuclear ribonucleoprotein A2B1	Exon 2	Skipping	Skipping	Y	Y – Koz et al., 1995	hnRNP B1	Y – Lung cancer - Sueoka et al., 1999; Kamma et al., 1999
LRRC23	Leucine-rich repeat-containing protein 23	Exon 3	Inclusion	Inclusion	Y	N	-	N
NRG1	Neuregulin 1	Exon 8	Skipping	Skipping	Y	?	?	? – NRG1 disregulation disrupts synaptic plasticity - Agarwal et al., 2014
HIF1α	Hypoxia-inducible factor 1-alpha	Exon 14	Inclusion	Inclusion	Y	Y - Gothie et al., 2000	$HIF1\alpha^{736}$ and sHIF1α	Y- higher expression levels of $HIF1\alpha^{736}$ in OR-negative carcinomas - Dales et al., 2010
FN1	Fibronectin	Exon 33	Skipping	Skipping	Y	Y - Goossens et al.,2009	EDA or EDI	Y – DM1 - Ohsawa et al., 2011
ACCN3	acid sensing (proton gated) ion channel 3	Exon 6	--	Inclusion	Y	?	?	N
CLCN6	Chloride Channel, Voltage-Sensitive 6	Exon 4	Inclusion	Skipping	Y	Y – Eggermont et al., 1997	CIC6b and Exon y+3	N

Y- Yes; N- No; ? - Variant reported in databases (NCBI) however could not find variant in publication; - - No information
